# Ordered Macro–Microporous ZIF-8 Decorated with Nanoparticles for Highly Sensitive Detection of Auramine O in Tropical Fruits

**DOI:** 10.3390/nano16070398

**Published:** 2026-03-25

**Authors:** Weiao Li, Litiao Ren, Yuqi Zhao, Xinping Cong, Mingjin Zhang, Yan Liu, Qihui Shen, Xiaoyang Liu

**Affiliations:** 1 College of Chemistry and Pharmaceutical Engineering, Jilin University of Chemical Technology, Jilin 132022, China; 2 State Key Laboratory of Inorganic Synthesis & Preparative Chemistry, Jilin University, Changchun 130021, China

**Keywords:** ordered macro–microporous MOF, electrochemistry, auramine O

## Abstract

Herein, an electrochemical sensor is reported for the first time based on an ordered macro–microporous composite derived from metal–organic frameworks (MOFs) for the highly sensitive detection of auramine O (AO), a Group 2B carcinogen. The hierarchical pore architecture, integrating an ordered macroporous network with a microporous ZIF-8 framework, enables the uniform dispersion of a high density of catalytically active sites. The interconnected macroporous channels facilitate efficient mass transport and rapid removal of reaction byproducts, effectively preventing pore blockage and ensuring stable sensing performance during repeated measurements. Owing to these structural advantages, the proposed sensor exhibits outstanding analytical performance toward AO detection, with a sensitivity of 0.4843 μA μM^−1^, a detection limit of 0.168 μM (S/N = 3), and a wide linear range from 0.5 to 50 μM. Moreover, the sensor demonstrates excellent selectivity and reproducibility, maintaining reliable responses even in the presence of 100-fold excess common food constituents such as tartrazine and glucose. Real sample analysis further confirms its high accuracy and operational stability. Overall, the electrochemical sensor based on silver nanoparticle-decorated ordered macro–microporous ZIF-8 synthesized via in situ reduction shows great potential as a portable and on-site tool for rapid AO detection in food. More broadly, ordered macro–microporous MOF-derived materials represent a promising platform for advanced electrochemical sensor applications.

## 1. Introduction

Auramine O (AO) is a synthetic diarylmethane dye that has been classified by the International Agency for Research on Cancer (IARC) as a Group 2B carcinogen [1]. Although its use as a food additive has been prohibited or not authorized in many countries and regions, including China, the European Union, Japan, and the United States, AO is still illegally added to some fruits and soy-based products because of its low cost and bright yellow color [2,3]. Therefore, the presence of AO in food is of regulatory concern and creates an urgent need for rapid, sensitive, and reliable analytical methods for food safety monitoring.

A variety of analytical methods have been developed for the determination of AO in food matrices. Li et al. reported a molecularly imprinted solid-phase extraction coupled with high-performance liquid chromatography (MISPE–HPLC) method, which achieved recoveries of 90.5–92.4% [4]. However, this approach involves complex in situ polymerization, multistep pretreatment, and relatively bulky instrumentation, which limit its practicality for rapid on-site analysis. Han et al. developed a carbon dot-enhanced chemiluminescence method with a low detection limit of 35 nM [5], but the associated flow system and instrument setup remain inconvenient for portable applications. Zhang et al. introduced a dynamic surface-enhanced Raman spectroscopy method for AO determination in soy products, with relative standard deviations below 8.3% during continuous use [6]. Nevertheless, the strict requirements for environmental stability and operation timing may restrict its routine use in food inspection. Other emerging approaches, including G-quadruplex-based fluorescence [7], terahertz spectroscopy [8], and smartphone-assisted fluorescent sensing [9], have also been explored.

Electrochemical methods have attracted increasing attention for food contaminant analysis because of their high sensitivity, rapid response, low cost, and compatibility with portable devices [10]. For AO determination, previous electrochemical sensors, such as adsorptive stripping voltammetry and nanocomposite-modified electrodes, have demonstrated the feasibility of rapid detection with relatively simple instrumentation [11,12]. However, these reported systems still present several limitations, including relatively narrow linear ranges, detection limits that can be further improved, and insufficient consideration of how electrode architecture affects analyte transport, active-site accessibility, and long-term sensing stability. More broadly, recent reviews on MOF-based electrochemical materials and sensors have pointed out that, despite their promise, such systems often remain limited by low intrinsic conductivity, restricted mass transport, and the incomplete utilization of internal active sites [13,14]. Therefore, the rational design of electrochemical sensing materials with both efficient mass transport and abundant electroactive sites remains highly desirable for improving AO detection performance.

Metal–organic frameworks (MOFs) are crystalline porous materials assembled from metal nodes and organic ligands [15]. Owing to their high surface area, tunable pore structures, and versatile chemical functionality, MOFs have been widely investigated in catalysis, separation, adsorption, and sensing [16]. In electrochemical sensing, MOFs are particularly attractive because they can provide large surface areas for analyte enrichment and abundant sites for the immobilization of catalytically active components [13]. Nevertheless, the practical performance of conventional microporous MOFs is often limited by low intrinsic conductivity, restricted analyte/electrolyte transport, and insufficient accessibility of internal active sites [14]. These characteristics reduce the effective utilization of the porous framework and may lead to sluggish sensor response and unsatisfactory electrocatalytic efficiency.

To overcome these drawbacks, hierarchically porous MOFs, especially ordered macro–microporous architectures, have been developed to integrate the adsorption capability of microporous frameworks with the rapid mass-transport characteristics of interconnected macroporous channels [17]. In such structures, the ordered macropores can facilitate electrolyte penetration, accelerate analyte diffusion to the electroactive interface, and promote the timely removal of reaction intermediates or byproducts, thereby alleviating diffusion resistance and helping maintain stable sensing performance during repeated measurements [18]. Meanwhile, the preserved microporous framework offers abundant adsorption and anchoring sites, which are beneficial for target enrichment and the uniform dispersion of catalytically active nanoparticles [19]. Therefore, compared with conventional microporous MOFs, ordered macro–microporous ZIF-8 is expected to provide higher active-site utilization, faster interfacial mass transport, and better operational stability, making it a more advantageous platform for electrochemical sensing [20].

Based on these considerations, we report herein an electrochemical sensor based on silver nanoparticle-decorated ordered macro–microporous ZIF-8 (Ag-SOM-ZIF-8) for the sensitive detection of AO. The hierarchical SOM-ZIF-8 framework provides a three-dimensional transport network and abundant anchoring sites for nanoparticle loading, while Ag nanoparticles improve electrical conductivity and electrocatalytic activity. Benefiting from the synergistic effects of the ordered macro–microporous architecture and uniformly dispersed Ag nanoparticles, the proposed Ag-SOM-ZIF-8/GCE sensor exhibits sensitive and reliable AO detection performance and shows promising potential for practical analysis in fruit samples.

## 2. Experimental

### 2.1. Chemicals

2-Methylimidazole (C_4_H_6_N_2_, 98%), zinc nitrate hexahydrate (Zn(NO_3_)_2_·6H_2_O, 99%), silver nitrate (AgNO_3_, 99.5%), sodium borohydride (NaBH_4_, 99%), and potassium persulfate (K_2_S_2_O_8_, 99%) were purchased from Aladdin Reagent Co., Ltd., Shanghai, China. Sodium hydroxide (NaOH, 99.5%), dimethyl sulfoxide (DMSO, (CH_3_)_2_SO, 98%), *N*-methyl-2-pyrrolidone (NMP, 99.5%), *N*,*N*-dimethylformamide (DMF, 99%), styrene (C_6_H_5_CH = CH_2_, 99%), and polyvinylpyrrolidone (PVP K30) were obtained from Innochem Reagent Co., Ltd., Beijing, China. Auramine O (C_17_H_22_ClN_3_, 75%), disodium hydrogen phosphate (Na_2_HPO_4_, 99%), and potassium dihydrogen phosphate (KH_2_PO_4_, 98%) were supplied by Macklin Reagent Co., Ltd., Shanghai, China. Anhydrous ethanol (CH_3_CH_2_OH, 99%), anhydrous methanol (CH_3_OH, 99.5%), and aqueous ammonia solution (NH_3_(aq), 70%) were provided by Sinopharm Chemical Reagent Co., Ltd., Shanghai, China. All chemicals used in this study were of analytical grade and were used as received without further purification. Deionized water was used as the solvent throughout all experimental procedures.

### 2.2. Characterization Methods

Scanning electron microscopy (SEM) images were obtained using a ZEISS Gemini SEM 300 (Carl Zeiss, Oberkochen, Germany). Before microscopy, each specimen was coated with an ultrathin Au film by sputtering for 45 s (Quorum SC7620 (Quorum Technologies Ltd., East Grinstead, UK)), and imaging was conducted at an accelerating voltage of 3 kV. Elemental composition was examined by energy-dispersive X-ray spectroscopy using an Oxford XPLORE30 system (Oxford Instruments, Abingdon, UK) operated at 15 kV, while spatial distribution of the elements was visualized through mapping collected with the SE2 secondary-electron detector. High-resolution transmission electron microscopy (HRTEM) and high-angle annular dark-field scanning transmission electron microscopy (HAADF-STEM) images were acquired on a JEOL JEM-2100F transmission electron microscope (JEOL Ltd., Tokyo, Japan). Fourier transform infrared (FTIR) spectra were recorded on a Thermo Fisher Nicolet iS20 spectrometer (Thermo Fisher Scientific, Waltham, MA, USA) using the KBr pellet method (sample/KBr ≈ 1:100). FTIR measurements were acquired from 4000 to 400 cm^−1^ using a spectral resolution of 4 cm^−1^ with 32 accumulated scans. Powder XRD was performed on a Rigaku Ultima IV instrument (Rigaku Corporation, Tokyo, Japan) with Cu Kα irradiation (λ = 1.5406 Å) at an operating voltage/current of 40 kV/40 mA. Diffraction profiles were collected between 2θ = 5° and 50°, using 0.02° increments and a scan speed of 2° min^−1^. XPS characterization was carried out using a Thermo Scientific K-Alpha system (Thermo Fisher Scientific, Waltham, MA, USA) fitted with a monochromated Al Kα excitation source (hν = 1486.6 eV). Wide-scan spectra were acquired at 1.0 eV energy intervals, whereas narrow-scan (high-resolution) regions were measured with a finer step of 0.1 eV.

### 2.3. Preparation of 3D Ordered PS Template

A three-dimensionally ordered polystyrene (PS) template was prepared via a classical emulsion polymerization followed by centrifugal self-assembly [13]. Briefly, purified styrene monomers were polymerized in boiling water containing polyvinylpyrrolidone (PVP) after deoxygenation. The resulting PS microspheres were purified by repeated centrifugation and redispersion, and subsequently assembled into an ordered structure by gradient centrifugation. The assembled material was dried at 70 °C, ground, and sieved to obtain a three-dimensionally ordered PS template with an opal-like structure.

### 2.4. Preparation of SOM-ZIF-8

The as-prepared PS template was vacuum-impregnated with a mixed precursor solution of dimethyl sulfoxide (DMSO) and ethanol containing Zn^2+^ and 2-methylimidazole. After vacuum drying at 45 °C to remove ethanol, a precursor–template composite was obtained. The composite was then vacuum-impregnated with a crystallization inducer consisting of methanol and aqueous ammonia (volume ratio = 1:6) and reacted at 30 °C for 12 h to form a MOF–template composite. Subsequently, the PS template was removed by treating the composite with N,N-dimethylformamide (DMF) three times, yielding SOM-ZIF-8. Finally, the product was thoroughly washed, vacuum-dried, and stored for further use [14].

### 2.5. Preparation of Ag-SOM-ZIF-8

Ag-SOM-ZIF-8 was synthesized by an impregnation–reduction strategy [17]. In a typical synthesis, SOM-ZIF-8 (0.05 g) was dispersed in 8 mL of an AgNO_3_ solution (10 mmol L^−1^) and maintained under stirring at ambient temperature for 6 h to load Ag^+^ onto the framework. The product was then isolated by centrifugation and vacuum-dried at 50 °C for 8 h to obtain the Ag^+^-loaded intermediate (Ag^+^/SOM-ZIF-8). Next, NaBH_4_ (20 mg) was dissolved in 4 mL of deionized water and introduced dropwise into the suspension while stirring vigorously (1200 rpm), converting Ag^+^ into metallic Ag nanoparticles as evidenced by the immediate color change. After 30 min of reaction, the composite was collected by centrifugation, thoroughly rinsed with deionized water, and finally dried under vacuum at 50 °C for 12 h.

### 2.6. Fabrication of the Ag-SOM-ZIF-8 Modified Electrode

A glassy carbon electrode (GCE, 3 mm in diameter; geometric surface area = 0.07 cm^2^) was used as the working substrate. Prior to modification, the GCE was sequentially polished with 0.3 and 0.05 μm alumina slurry, thoroughly rinsed with deionized water, and ultrasonically cleaned in ethanol for 1 min. The cleaned electrode was then electrochemically activated by performing 20 cyclic voltammetry scans in 0.5 M H_2_SO_4_ over the potential range from −0.2 to +1.0 V (vs. Ag/AgCl) at a scan rate of 100 mV s^−1^.

To prepare the catalyst suspension, 5 mg of Ag-SOM-ZIF-8 powder was dispersed in 5 mL of deionized water, followed by magnetic stirring for 30 min and ultrasonication for another 30 min to obtain a homogeneous suspension with a concentration of 1 mg mL^−1^. Subsequently, 10 μL of the suspension was drop-cast onto the surface of the activated GCE. After that, 2 μL of diluted Nafion solution (0.02 wt%) was applied as a thin binding/protective overlayer to improve film adhesion and mechanical stability while minimizing blockage of electroactive sites and diffusion pathways. The modified electrode was then dried at ambient conditions for 30 min before electrochemical measurements.

### 2.7. Electrochemical Measurements

Electrochemical measurements were carried out using a CHI 760E electrochemical workstation (CH Instruments, Shanghai, China) with a conventional three-electrode configuration. The Ag-SOM-ZIF-8 modified GCE served as the working electrode, a platinum wire as the counter electrode, and an Ag/AgCl electrode (saturated KCl) as the reference electrode. Cyclic voltammetry (CV) was performed over a potential range of −0.2 to +0.6 V at scan rates from 5 to 200 mV s^−1^. Electrochemical impedance spectroscopy (EIS) measurements were performed using a 5 mV AC perturbation over the frequency range from 10^5^ to 10^−2^ Hz. Unless otherwise specified, electrochemical tests were carried out at 25 °C in 0.1 M KCl containing 5 mM [Fe(CN)_6_]^3−^/^4−^ as the redox probe. Under the optimized experimental conditions, DPV was employed for the quantitative determination of auramine O. The DPV measurements were carried out with a pulse amplitude of 50 mV, a potential step of 4 mV, a pulse time of 50 ms, a pulse period of 0.20 s, a sampling width of 10 ms, and a quiet time of 3 s. The potential scan was performed in the positive-going direction, corresponding to an effective scan rate of 20 mV s^−1^. These parameters were selected based on optimization experiments to achieve a sensitive and stable electrochemical response.

## 3. Results and Discussion

### 3.1. Characterization

**Figure 1:**
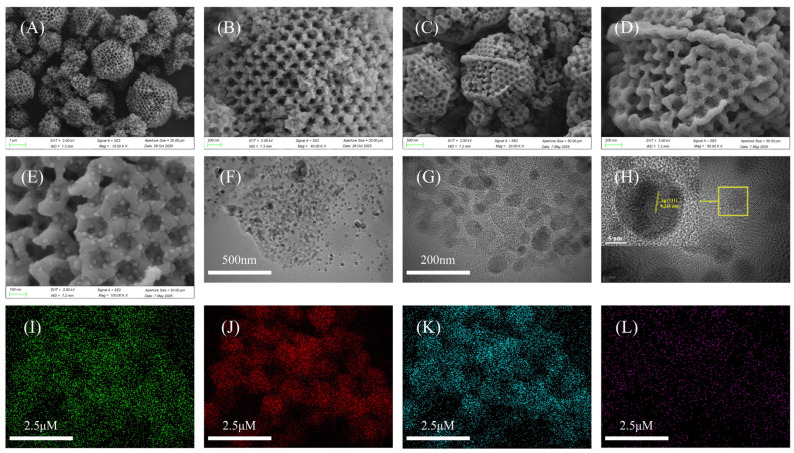
Morphological and structural characterization of ZIF-8–based composites. (**A**,**B**) SEM images of SOM-ZIF-8. (**C**–**E**) SEM images, (**F**) HAADF-STEM image, and (**G**,**H**) HRTEM images of Ag-SOM-ZIF-8. (**I**–**L**) EDS elemental mapping of Ag-SOM-ZIF-8.

SEM images (Figure 1A,B) reveal that SOM-ZIF-8 exhibits a well-defined ordered macroporous structure on its surface, with macropores of approximately 160 nm in diameter and particle sizes of about 1.2 μm. This highly ordered macroporous architecture is consistent with the inverse opal structure replicated from the PS template, confirming the successful template-guided growth process. Ag nanoparticles (Ag NPs) were successfully introduced into SOM-ZIF-8 via the impregnation–reduction method. As shown in Figure 1C–E, Ag-SOM-ZIF-8 retains the characteristic tetrahedral single-crystal morphology of the parent SOM-ZIF-8, indicating that the incorporation of Ag NPs does not disrupt the crystallinity of the MOF framework, even at relatively high Ag loadings. The Ag NPs, with an average diameter of approximately 10 nm, are homogeneously distributed within the macroporous channels as well as on the external surface. HRTEM images (Figure 1F,G) further confirm the uniform dispersion of Ag NPs throughout the SOM-ZIF-8 matrix. As shown in Figure 1H, distinct lattice fringes are visible, corresponding to a d-spacing of 0.236 nm, corresponding to the (111) plane of face-centered cubic (fcc) silver. EDS elemental mapping images (Figure 1I–L) demonstrate the homogeneous distribution of Ag across the entire MOF structure. The signals of C, N, and Zn originate from the ZIF-8 framework, while the Ag signal is attributed to the incorporated nanoparticles. Quantitative EDS analysis indicates mass fractions of 5.12% for Ag and 1.44% for Zn. Notably, the ordered macroporous structure of SOM-ZIF-8 remains well preserved after Ag NP loading, and no significant nanoparticle aggregation is observed, highlighting the structural robustness and effective confinement capability of the inverse opal MOF architecture.

**Figure 2:**
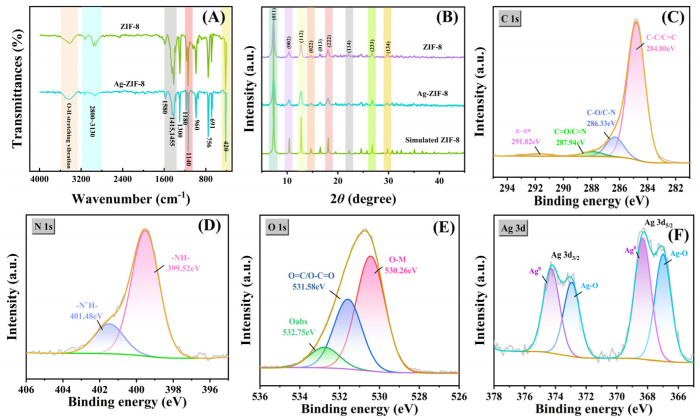
Structural and chemical characterization of ordered macro–microporous ZIF-8 composites. (**A**) FTIR spectra. (**B**) XRD patterns. (**C**–**F**) High-resolution XPS spectra of Ag-SOM-ZIF-8: C 1s (**C**), N 1s (**D**), O 1s (**E**), and Ag 3d (**F**).

The Fourier transform infrared (FT-IR) spectrum of SOM-ZIF-8 (Figure 2A) exhibits a series of characteristic absorption bands at approximately 420, 690, 755, 1140, 1180, 1305, 1415, 1455, and 1580 cm^−1^, which are consistent with the typical vibrational features of ZIF-8. The band at around 420 cm^−1^ can be assigned to the Zn–N stretching vibration coupled with the out-of-plane twisting mode of the imidazole ring [19]. The absorption peaks at 690 and 755 cm^−1^ originate from the out-of-plane bending vibrations of the C–H bonds in the imidazole ring. The bands observed at 1140 and 1180 cm^−1^ correspond to C–N stretching vibrations [20]. In addition, the band located at 1305 cm^−1^ can be assigned to the ring-stretching mode of the imidazole framework as a whole. The absorption bands at 1415 and 1455 cm^−1^ are associated with C=N and C=C stretching vibrations within the imidazole ring, whereas the peak at 1580 cm^−1^ is assigned to the symmetric stretching of C=N and C=C bonds [21]. Moreover, a wide band spanning 2800–3130 cm^−1^ arises from C–H stretching modes associated with the methyl group and aromatic moieties of 2-methylimidazole [22]. The FT-IR spectrum of Ag-SOM-ZIF-8 shows the same characteristic absorption features as SOM-ZIF-8, indicating that the framework structure remains intact after Ag nanoparticle incorporation [23]. Notably, the overall intensity of the absorption bands is reduced, which is commonly attributed to the infrared shielding or scattering effect induced by the presence of metal nanoparticles within the pores or on the surface of the MOF. The X-ray diffraction (XRD) pattern of SOM-ZIF-8 (Figure 2B) displays sharp diffraction peaks at 2θ values of 7.3°, 10.4°, 12.7°, 14.7°, 16.4°, and 18.0°, which are well indexed to the sodalite topology of ZIF-8. After Ag loading, all characteristic diffraction peaks of SOM-ZIF-8 are preserved in the Ag-SOM-ZIF-8 sample, demonstrating that the crystalline framework remains stable during the impregnation–reduction process [24]. Notably, no diffraction peaks corresponding to face-centered cubic (fcc) Ag (typically at 38.1° and 44.3°) are detected [24]. The lack of detectable Ag reflections is likely due to the very small particle size (rendering them XRD-invisible) and/or attenuation of diffraction by a thin, amorphous Ag_2_O layer on the nanoparticle surface, in agreement with the TEM and XPS observations. X-ray photoelectron spectroscopy (XPS) was employed to further investigate the chemical states and interfacial interactions in Ag-SOM-ZIF-8 (Figure 2C–F). Deconvolution of the C 1s spectrum (Figure 2C) reveals three components centered at 284.80 eV (C–C/C=C in the imidazole ring), 286.33 eV (C–O/C–N), and 287.94 eV (C=N). The pronounced C=N peak confirms the structural integrity of the 2-methylimidazole ligand, while the satellite peak at 291.82 eV is characteristic of the conjugated aromatic system [25]. The N 1s spectrum (Figure 2D) exhibits a dominant peak at 399.52 eV, corresponding to pyridinic-type nitrogen (–NH–) in the imidazole ring [26]. The O 1s spectrum (Figure 2E) can be deconvoluted into three components located at 530.26 eV (O–M, M = Zn/Ag, associated with hydroxylated metal nodes or Ag–O species), 531.58 eV (O–C=O from adsorbed species), and 533.20 eV (physically adsorbed H_2_O) [27]. Since the ideal ZIF-8 framework itself does not contain oxygen atoms, the observed O 1s signal is attributed mainly to surface-associated oxygen species rather than to the framework structure [18]. Specifically, these oxygen species may arise from physically adsorbed H_2_O, oxygen-containing adsorbates, hydroxylated surface metal sites, and minor Ag–O/Ag_2_O-related species formed upon air exposure. The Ag 3d spectrum (Figure 2F) shows two characteristic peaks at 368.0 eV (Ag 3d_5/2_) and 374.0 eV (Ag 3d_3/2_), confirming the presence of metallic silver nanoparticles. Further peak fitting reveals that Ag^0^ is the dominant species, accompanied by a minor contribution from Ag_2_O (367.3/373.3 eV), suggesting the formation of a thin surface oxide layer due to exposure to air [28].

### 3.2. Electrochemical Performance Evaluation of Ag-SOM-ZIF-8

**Figure 3:**
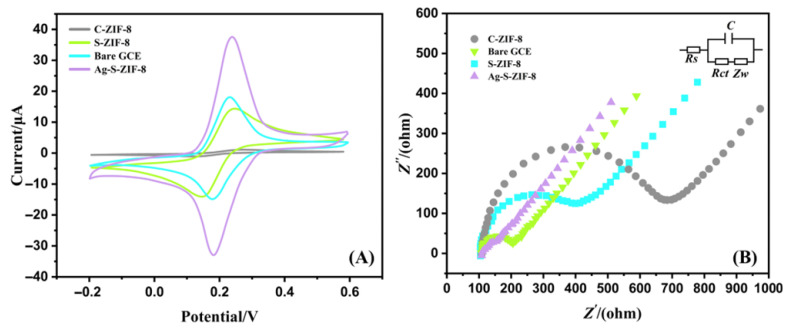
Cyclic voltammetry (CV) (**A**) and electrochemical impedance spectroscopy (EIS) analyses ((**B**), inset: equivalent electrical circuit) responses of Ag-SOM-ZIF-8/GCE, SOM-ZIF-8/GCE, C-ZIF-8/GCE, and bare GCE in 0.1 M KCl containing 5 mM [Fe(CN)_6_]^3−^/^4−^.

Cyclic voltammetry (CV) measurements were carried out in 0.1 M KCl containing 5 mM [Fe(CN)_6_]^3−^/^4−^ to evaluate the electrochemical reactivity of different modified electrodes, including Ag-SOM-ZIF-8/GCE, SOM-ZIF-8/GCE, microporous ZIF-8/GCE (denoted as C-ZIF-8/GCE), and the bare GCE. As shown in Figure 3A, well-defined redox peaks were observed for Ag-SOM-ZIF-8/GCE, SOM-ZIF-8/GCE, and the bare GCE, whereas only negligible currents were detected for C-ZIF-8/GCE. Among all electrodes, Ag-SOM-ZIF-8/GCE exhibited the highest redox peak current of 37.72 μA, which is markedly higher than that of SOM-ZIF-8/GCE (14.76 μA) and the bare GCE. Although ZIF-8 frameworks generally possess limited intrinsic electrical conductivity, the three-dimensionally ordered macroporous structure of SOM-ZIF-8 significantly enhances electrolyte accessibility and mass transport, thereby improving electrochemical performance [29]. In contrast, the microporous structure of conventional ZIF-8 (C-ZIF-8) imposes severe diffusion constraints, restricting electrochemical reactions to a thin interfacial layer due to size-exclusion effects. These results demonstrate that SOM-ZIF-8 provides a more favorable scaffold for hosting electroactive species, enabling superior electrochemical activity compared to its microporous counterpart [30]. The electrochemically active surface areas (ECSAs) of the electrodes were estimated using the Randles–Ševčík equation. The calculated ECSAs followed the order: Ag-SOM-ZIF-8 (0.0686 cm^2^) > SOM-ZIF-8 (0.0298 cm^2^) > bare GCE (0.0324 cm^2^), confirming the pronounced surface activation effect induced by the hierarchical porous architecture and Ag nanoparticle incorporation [31]. Electrochemical impedance spectroscopy (EIS) was employed to further elucidate the interfacial charge transfer and mass transport behavior of the electrodes. Nyquist plots were recorded in 0.1 M KCl containing 5 mM [Fe (CN)_6_]^3−^/^4−^ for Ag-SOM-ZIF-8/GCE, SOM-ZIF-8/GCE, C-ZIF-8/GCE, and the bare GCE. As shown in Figure 3B, the semicircle in the high-frequency region corresponds to the charge-transfer resistance (*R_ct_*), while the inclined line in the low-frequency region is associated with diffusion-controlled processes. The experimental EIS data were fitted using a Randles equivalent circuit consisting of a solution resistance (*R_s_*), a charge-transfer resistance (*R_ct_*), a constant phase element (CPE), and a Warburg impedance (*Z_W_*) [32]. The fitted impedance parameters are summarized in Table S1. Among the four electrodes, Ag-SOM-ZIF-8/GCE exhibits the lowest charge-transfer resistance (*R_ct_* = 152.36 Ω), which is markedly smaller than those of bare GCE (224.3 Ω), SOM-ZIF-8/GCE (359.5 Ω), and C-ZIF-8/GCE (668.5 Ω), indicating that the incorporation of Ag nanoparticles together with the ordered macro–microporous framework greatly facilitates interfacial electron transfer. In addition, the solution resistance (*R_s_*) of Ag-SOM-ZIF-8/GCE (52.66 Ω) is comparable to that of bare GCE (54.23 Ω) and clearly lower than those of SOM-ZIF-8/GCE (84.25 Ω) and C-ZIF-8/GCE (105.67 Ω), suggesting reduced overall ohmic resistance and improved electrolyte/electrode contact. The constant phase element parameters further support the favorable interfacial characteristics of Ag-SOM-ZIF-8/GCE. Specifically, Ag-SOM-ZIF-8/GCE shows the highest Q value (1.86 × 10^−5^), implying a relatively large effective interfacial capacitance, which may be associated with its larger electrochemically accessible surface and more active interface. Its *n* value (0.91) is also close to 1, indicating a near-ideal capacitive behavior and a relatively homogeneous interfacial structure. In contrast, C-ZIF-8/GCE shows the lowest *n* value (0.72), suggesting a more heterogeneous and less ideal interface, consistent with its severe diffusion limitation and poor charge-transfer capability. Overall, the fitted EIS results agree well with the CV results and confirm that the synergistic combination of the ordered macro–microporous architecture and Ag nanoparticles effectively enhances both charge transfer and interfacial electrochemical properties [33]. These features are critical contributors to the superior electrocatalytic performance of Ag-SOM-ZIF-8/GCE for auramine O detection.

### 3.3. Electrochemical Performance of Ag-SOM-ZIF-8 Toward Auramine O (AO)

**Figure 4:**
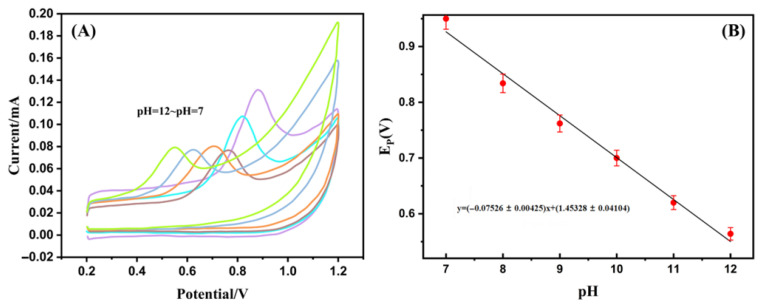
(**A**) Cyclic voltammogram of Ag-SOM-ZIF-8/GCE recorded in phosphate-buffered solution containing 50 μM AO. (**B**) Dependence of oxidation peak parameters on pH, obtained by curve fitting.

The electrochemical response of the Ag-SOM-ZIF-8 modified electrode was first evaluated as a function of modifier loading. Among the tested concentrations, the electrode prepared with a modifier concentration of 1 mg mL^−1^ exhibited the highest current response toward AO and was therefore selected for subsequent investigations. The effect of solution pH on the electrochemical behavior of AO was investigated using Ag-SOM-ZIF-8/GCE over the pH range of 5.0–12.0. As shown in Figure 4A, the voltammetric response of AO strongly depends on the solution pH. The peak current reaches its maximum at pH 7.0 and gradually decreases as the pH increases from 7.0 to 12.0. When the pH was further decreased to 6.0 and 5.0, the AO-related signal was markedly suppressed and the oxidation peak became indistinct, indicating that acidic conditions are unfavorable for AO detection on Ag-SOM-ZIF-8/GCE. A linear relationship between the peak potential and pH was obtained in the applicable pH range (Figure 4B), suggesting the involvement of proton-coupled electron transfer in the electrochemical process [34]. The signal suppression under acidic conditions is likely due to the combined effects of enhanced protonation of AO and reduced structural/surface stability of the ZIF-8-based sensing interface in acidic media. Therefore, pH 7.0 was selected as the optimal working condition for subsequent measurements because it provided the strongest and most stable response [35,36]. A linear relationship between *E_pc_* and pH was obtained (Figure 4B), which can be expressed as:

(1) $$E_{p}=\left(-0.07526\pm 0.00425\right)\mathrm{pH}+\left(1.45328\pm 0.04104\right)\left(R^{2}=0.991\right)$$

The slope of −0.07526 V pH^−1^ is close to the theoretical Nernstian value of −0.059 V pH^−1^, indicating that the electrochemical reaction involves an equal number of protons and electrons (m = n). The gradual decrease in I_pc with increasing pH is likely attributed to weakened adsorption of AO under alkaline conditions. Moreover, the disappearance of the oxidation peak at pH < 7.0 may result from protonation-induced deactivation of active sites, while framework instability of ZIF-8 under strongly alkaline conditions (pH > 12) may also contribute to signal loss [36]. Consequently, the optimal working pH range was determined to be 7.0–12.0, with pH 7.0 selected for subsequent measurements due to the highest signal intensity.

**Figure 5:**
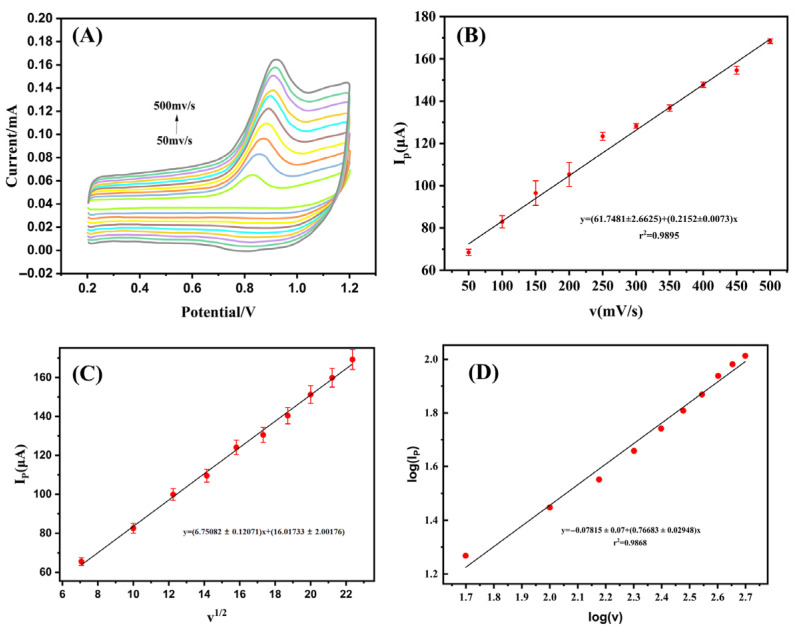
(**A**) Cyclic voltammograms of Ag-SOM-ZIF-8/GCE recorded in 0.1 M phosphate-buffered solution (pH 7.0) containing 50 μM auramine O (AO) at different scan rates. (**B**) Corresponding voltammetric responses at varying scan rates. (**C**) Linear relationship between the oxidation peak current (*I_P_*) and the square root of the scan rate (*v*^1/2^). (**D**) Linear relationship between log *I_P_* and log *v.*

The influence of scan rate on the electrochemical behavior of AO at Ag-SOM-ZIF-8/GCE was investigated to clarify the kinetic characteristics of the oxidation process (Figure 5). As the scan rate increased, the oxidation peak current increased accordingly, while the peak potential shifted gradually toward more positive values, indicating the kinetically irreversible nature of the AO oxidation process [37].

As shown in Figure 5B,C, the oxidation peak current *I_P_* exhibited linear relationships with both the scan rate (*v*) and the square root of the scan rate (*v*^1/2^). Since both relationships showed acceptable linearity, the control mechanism could not be reliably assigned on the basis of these two plots alone. Therefore, a log (*I_P_*) versus log (*v*) analysis was further performed according to the power-law relationship *I_P_* = *av^b^*. The fitted equation was:

(2) $$I_{p}(\mu A)=(0.2152\pm 0.0073)v({\mathrm{mV}\;s}^{-1})+(61.7481\pm 2.6625)(R^{2}=0.9895)$$

(3) $$I_{p}(\mu A)=(6.75082\pm 0.12071)v^{1/2}[({\mathrm{mV}\;s}^{-1}{)}^{1/2}]+(16.01733\pm 2.00176)(R^{2}=0.9923)$$

(4) $$logI_{P\;}=-0.07815+0.76683\log v\;(R^{2}=0.9868)$$

The slope value (*b* = 0.76683) indicates that the electrochemical oxidation of AO at Ag-SOM-ZIF-8/GCE follows a mixed adsorption–diffusion-controlled process. Because the reaction is not an ideal surface-confined process, the direct extraction of a definitive heterogeneous electron-transfer rate constant or exact electron-transfer number using the simplified Laviron treatment is not sufficiently rigorous in the present system. Therefore, no reliable *k_s_* value is assigned here, and the previously estimated apparent electron-transfer number is not further interpreted as conclusive evidence for a specific six-electron oxidation pathway.

Combined with the pH-dependent behavior, the present results suggest that AO oxidation on Ag-SOM-ZIF-8/GCE is a proton-coupled, kinetically irreversible, multi-electron electrochemical process. Previous studies have shown that AO electro-oxidation may proceed through multiple oxidative steps and generate quinone/hydroquinone-related intermediates as well as further degradation products, rather than following a simple single-step pathway. Therefore, the present electrochemical results are more reasonably interpreted as evidence of a complex multi-step oxidation process, while the exact molecular pathway would require further mechanistic verification by complementary product analysis.

### 3.4. Linear Range, Detection Limit, and Practical Performance

**Figure 6:**
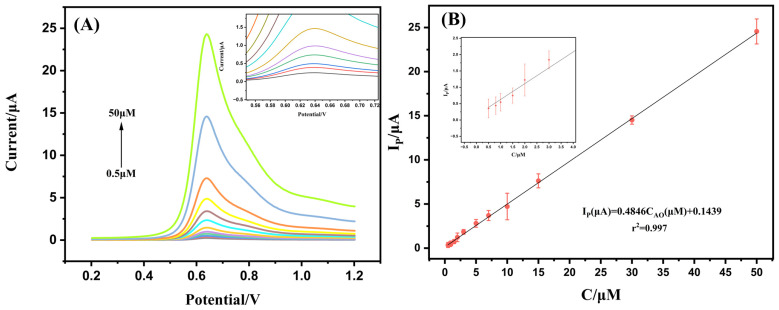
(**A**) Differential pulse voltammetry (DPV) responses of Ag-SOM-ZIF-8/GCE toward different concentrations of AO in 0.1 M PBS (pH 7.0). (**B**) Corresponding calibration plot of peak current versus AO concentration. Inset: Enlarged view of the low-concentration region, in which 0.5 μM represents the lower boundary of the practical calibration range and was further validated by recovery experiments.

Under optimized experimental conditions, the analytical performance of the Ag-SOM-ZIF-8/GCE sensor toward AO was evaluated using differential pulse voltammetry (DPV). As shown in Figure 6, the oxidation peak current exhibits a good linear relationship with AO concentration in the range from 0.5 to 50 μM, which can be expressed by the following regression equation:

(5) $$I_{p}(\mu A)=0.4846C_{\mathrm{AO}}(\mu M)+0.1439(R^{2}=0.997)$$

Although the 0.5 μM point is located at the lower boundary of the calibration range and shows slight visual deviation from the fitted line in the low-concentration inset, additional recovery experiments performed at this concentration yielded recoveries of 95.3–106.2%, confirming its acceptable quantitative applicability. Therefore, 0.5 μM was retained as the practical lower boundary of the linear working range.

Using an S/N criterion of 3, the detection limit was evaluated to be 0.168 μM. As summarized in Table 1, the analytical performance achieved in this work is competitive among reported electrochemical AO detection methods. Although the LOD of the present sensor is not lower than that of some fluorescence-based laboratory methods, auramine O is not an approved food colorant in major regulatory jurisdictions and is generally regarded as an unauthorized or prohibited dye in food rather than as a permitted additive with a harmonized maximum residue limit [1]. Therefore, the primary analytical requirement for food-safety monitoring is the reliable screening of illegal adulteration. In this regard, the present electrochemical sensor provides sufficient sensitivity for practical AO detection in food, while also offering the advantages of simple operation, rapid response, low cost, and potential portability.

**Figure 7:**
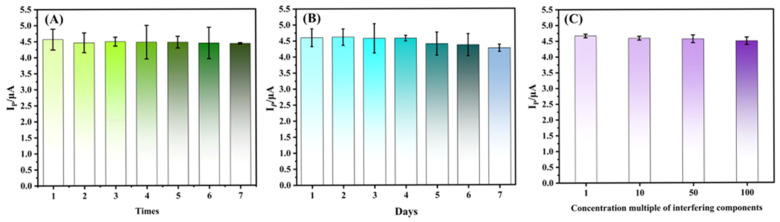
(**A**) Peak current (*I_p_*) responses for the continuous determination of auramine O. (**B**) DPV responses of auramine O recorded over seven consecutive days. (**C**) DPV responses in the presence of potential interfering species at different concentrations.

The reproducibility of the sensor was evaluated by repeatedly measuring 10 μM AO using the same Ag-SOM-ZIF-8/GCE over seven consecutive DPV cycles. After each measurement, the electrode was rinsed with PBS to remove surface-adsorbed species. As shown in Figure 7A, the peak current decreased by only 2.21% after seven consecutive measurements, indicating excellent reproducibility. This stable response can be attributed to the hierarchical porous structure, which facilitates efficient diffusion of reactants and mitigates blockage by reaction intermediates or products, as well as to the thin Nafion overlayer that helps maintain the integrity and adhesion of the catalyst film during repeated measurements [38].

The storage stability of the sensor was further investigated by storing the modified electrode in the dark at room temperature for seven days. The initial current response was obtained from triplicate measurements of 10 μM AO on day 0. As shown in Figure 7B, the current response retained 92.41% of its initial value after one week, corresponding to a minor signal loss of 7.59%. One possible explanation for this decrease is the mild surface oxidation/corrosion of Ag nanoparticles during storage in air. Such behavior has been reported for Ag-based nanostructures, for which ambient-air exposure may induce surface chemical changes and attenuate analytical performance [39]. Nevertheless, this explanation remains a plausible hypothesis in the present work and would require direct post-storage surface characterization, such as XPS, for confirmation.

Selectivity was evaluated in the presence of potential interfering substances commonly found in food matrices, including glucose, benzoic acid, citric acid, Na^+^, K^+^, tartrazine, and quinoline yellow. The current response of 10 μM AO was recorded in the presence of a 100-fold excess of each interferent. As shown in Figure 7C, none of the tested species caused an obvious change in the AO signal, indicating good selectivity and anti-interference capability of the sensor in a complex sample environment.

To evaluate practical applicability, the Ag-SOM-ZIF-8/GCE sensor was used for AO determination in spiked fruit samples (durian and jackfruit). The samples were diluted tenfold with the supporting electrolyte (0.1 M PBS, pH 7.0), and known concentrations of AO standard were added to the supernatants for recovery experiments. Each sample was analyzed using both the proposed electrochemical sensor and a reference HPLC method. As summarized in Table 2, the recoveries ranged from 98.1% to 105.9%, with relative standard deviations below 3.8%, demonstrating good accuracy and reliability of the proposed sensor for real-sample analysis.

## 4. Conclusions

In summary, an enzyme-free electrochemical sensor based on silver nanoparticle-loaded ordered macro–microporous ZIF-8 (Ag-SOM-ZIF-8/GCE) was successfully developed for the sensitive detection of auramine O. The hierarchical SOM-ZIF-8 framework provides abundant anchoring sites and a three-dimensional transport network, while the incorporated Ag nanoparticles improve electrical conductivity and electrocatalytic activity. Benefiting from the synergistic effects of the ordered macro–microporous architecture and the uniformly dispersed Ag nanoparticles, the Ag-SOM-ZIF-8/GCE sensor exhibited a linear detection range of 0.5–50 μM, a detection limit of 0.168 μM, and a sensitivity of 0.4843 μA μM^−1^, together with good selectivity, satisfactory reproducibility, and acceptable storage stability.

Electrochemical studies suggest that AO oxidation on Ag-SOM-ZIF-8/GCE is a proton-coupled, kinetically irreversible, multi-electron electrochemical process with mixed adsorption–diffusion characteristics. The improved sensing performance is mainly attributed to the enhanced interfacial charge transfer and mass transport enabled by the hierarchical porous structure and Ag nanoparticle incorporation. In addition, the proposed sensor showed reliable performance in spiked fruit samples, with recoveries of 98.1–105.9% and relative standard deviations below 3.8%, demonstrating its practical applicability for AO analysis in food matrices.

Overall, this work presents a robust and promising electrochemical platform for the rapid screening of illegal auramine O adulteration in food. Future work will focus on further improving long-term material stability and integrating the sensing system into portable analytical devices for on-site applications.

## Figures and Tables

**Table 1:** Performance comparison between this work and other advanced detection methods.

Method	Sensitivity	Linear Range (μM)	LOD (μM)	Refs.
UPLC-MS/MS	-	-	0.1	[1]
G-quadruplex-FS	-	0~0.07	0.0003	[6]
MISPE-HPLC	-	0.89~89.29	0.0625	[3]
ASV	0.246 µA·µM^−1^	0.4~4	0.248	[9]
DPV	2.0 µA·µM^−1^	1.9~17.0	0.65	[10]
This work	0.4843 µA·µM^−1^	0.5~50	0.168	-

**Table 2:** Recovery tests of AO in spiked fruit samples using the proposed sensor and HPLC method (n = 3).

Sample	Spiked Conc.	Sensor (μM)	HPLC (μM)	Recovery (%)	RSD (%)
Durian	0.5	0.49 ± 0.02	-	98.1	2.1
	2.0	2.06 ± 0.08	-	102.5	3.2
	10.0	10.32 ± 0.41	10.25 ± 0.30	103.2	3.5
Jackfruit	0.5	0.52 ± 0.02	-	104.1	2.8
	2.0	1.97 ± 0.07	-	98.5	3.1
	10.0	10.59 ± 0.42	10.55 ± 0.38	105.9	3.8
Banana	0.5	0.55 ± 0.03	-	110.0	5.45
	2.0	1.97 ± 0.11	-	98.5	5.58
	10.0	9.82 ± 0.42	10.13 ± 0.21	98.2	4.28-

## Data Availability

The original contributions presented in this study are included in the article/Supplementary Materials. Further inquiries can be directed to the corresponding authors.

## Supplementary Materials

The following supporting information can be downloaded at: https://www.mdpi.com/article/10.3390/nano16070398/s1, Figure S1. SEM image of the surface morphology of the 3D ordered polystyrene (PS) template and Cross-sectional SEM image of the 3D ordered polystyrene (PS) template. Figure S2. XPS survey spectrum of Ag-SOM-ZIF-8. Figure S3. Cyclic voltammetry (CV) responses of Ag-SOM-ZIF-8/GCE recorded in phosphate buffer solutions containing 50 μM auramine O at pH 6.0, 5.0, and 13.0. Figure S4. Differential pulse voltammetry (DPV) responses of Ag-SOM-ZIF-8/GCE toward auramine O at different accumulation times (0, 15, 30, and 60 s), recorded for optimization of the experimental conditions. Figure S5. Differential pulse voltammograms of Ag-SOM-ZIF-8/GCE prepared with different drop-cast volumes of the modifier suspension (5.0, 7.5, and 10.0 μL), recorded for optimization of the electrode-fabrication conditions. Table S1. Fitted EIS parameters of different electrodes based on the Randles equivalent circuit.
