# Supplementary material for: Ordered Macro–Microporous ZIF-8 Decorated with Nanoparticles for Highly Sensitive Detection of Auramine O in Tropical Fruits
Source: Nanomaterials (Basel). 2026 Mar 25;16(7):398. doi: 10.3390/nano16070398 (PMC13074856; doi:10.3390/nano16070398)
Supplement: Supplementary file 1 [file nanomaterials-16-00398-s001.zip › nanomaterials-4197970-supplementary.pdf]

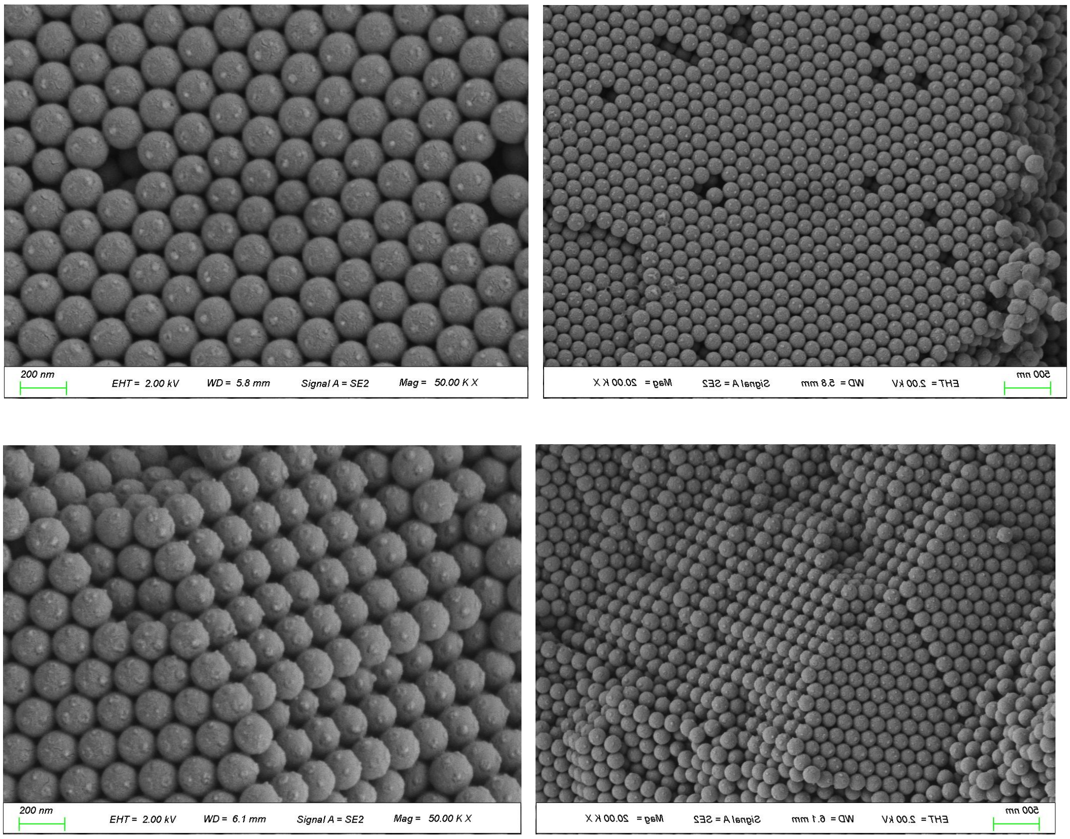

Figure S1. SEM image of the surface morphology of the 3D ordered polystyrene (PS) template and Cross-sectional SEM image of the 3D ordered polystyrene (PS) template.

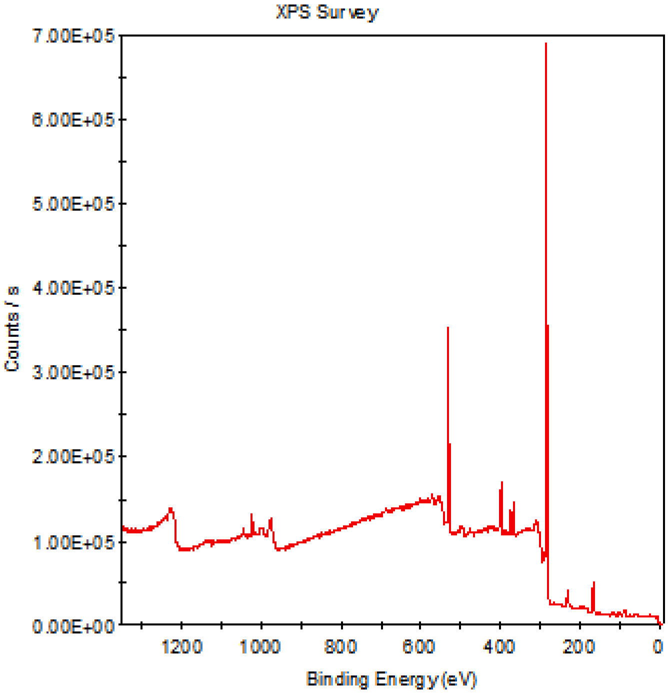

Figure S2. XPS survey spectrum of Ag-SOM-ZIF-8.

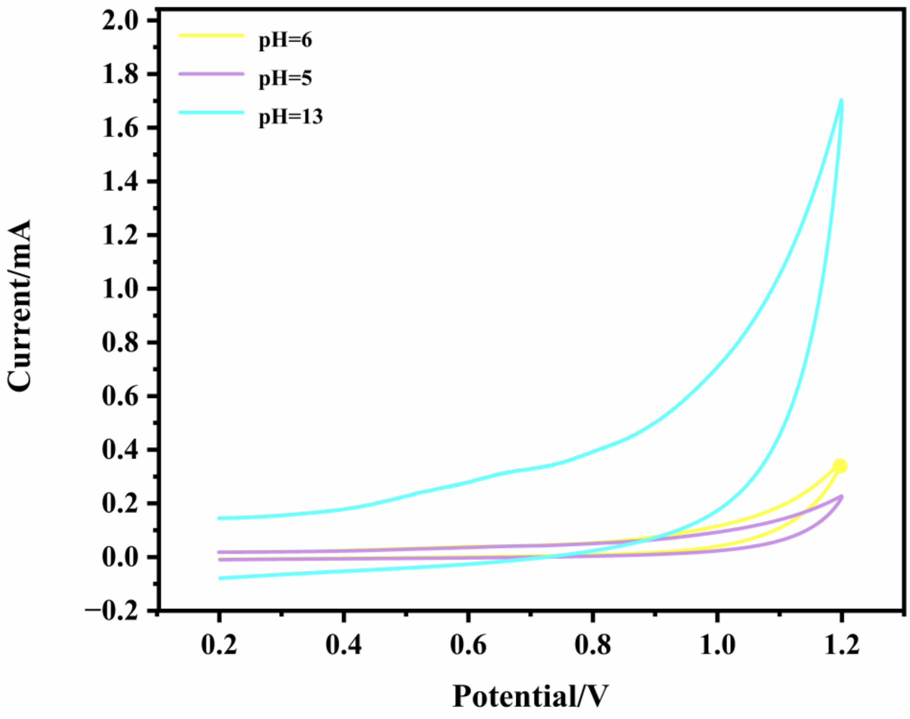

Figure S3. Cyclic voltammetry (CV) responses of Ag-SOM-ZIF-8/GCE recorded in phosphate buffer solutions containing 50 μM auramine O at pH 6.0, 5.0, and 13.0

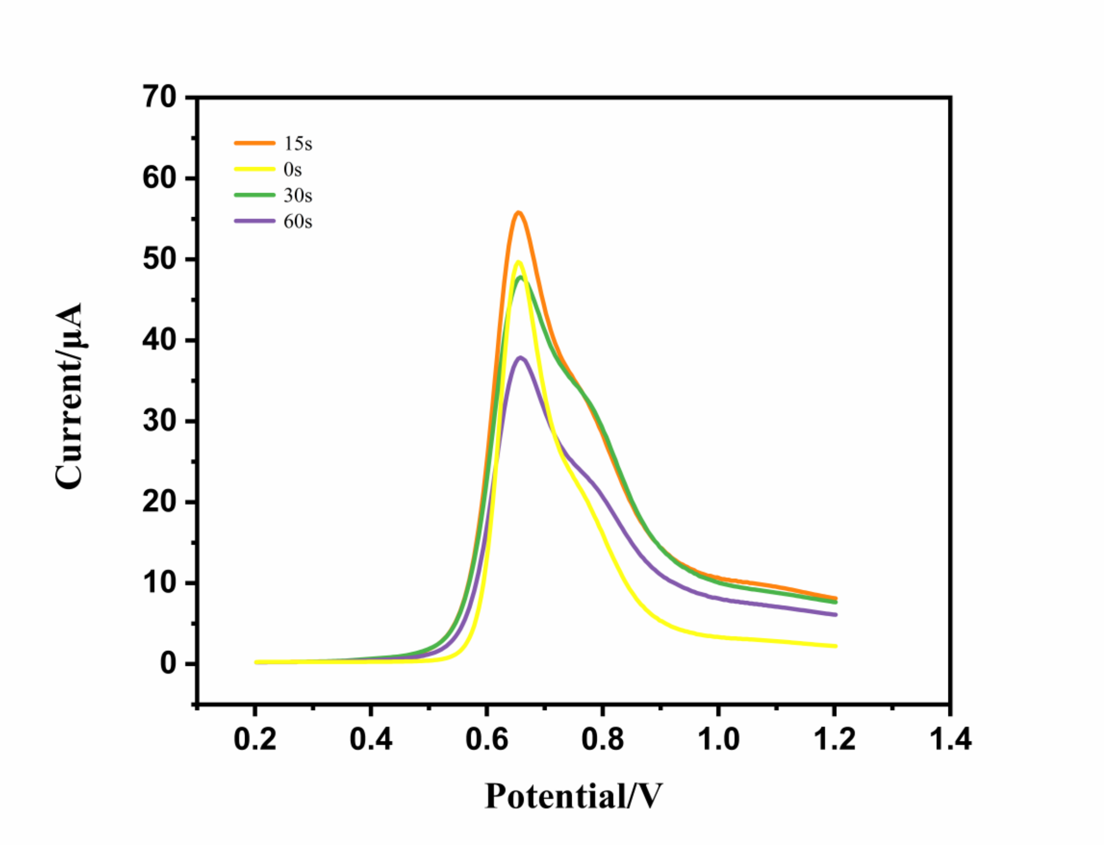

Figure S4. Differential pulse voltammetry (DPV) responses of Ag-SOM-ZIF-8/GCE toward auramine O at different accumulation times (0, 15, 30, and 60 s), recorded for optimization of the experimental conditions.

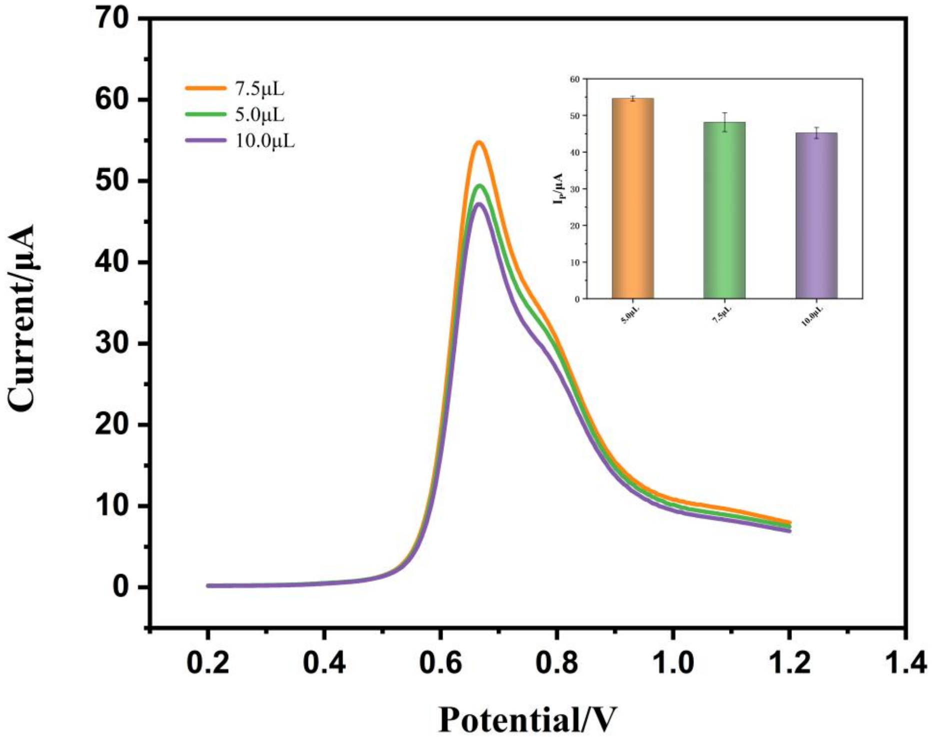

Figure S5. Differential pulse voltammograms of Ag-SOM-ZIF-8/GCE prepared with different drop-cast volumes of the modifier suspension (5.0, 7.5, and 10.0 μL), recorded for optimization of the electrode-fabrication conditions.\

Table S1. Fitted EIS parameters of different electrodes based on the Randles equivalent circuit.

**Electrode.**	R_s **/ Ω**	R_{ct} **/ Ω**	**CPE (** Q **)**	**CPE (** n **)**
Ag-SOM-ZIF-8/GCE	52.66	152.3	1.86×10^{-5}	0.91
bare GCE	54.23	224.3	0.56×10^{-5}	0.93
SOM-ZIF-8/GCE	84.25	359.5	1.02×10^{-5}	0.88
C-ZIF-8/GCE	105.67	668.5	0.88×10^{-5}	0.72
